# Nibbling 405 kb off the X: Viable deletion alleles eliminating 50 protein coding genes, including a chromatin factor involved in neuronal development

**DOI:** 10.17912/micropub.biology.000187

**Published:** 2019-10-25

**Authors:** Gregory Minevich, Alex Bernstein, Kevin Mei, Richard J Poole, Oliver Hobert

**Affiliations:** 1 Department of Biochemistiry and Molecular Biophysics, Columbia University; 2 present address: Department of Cell and Developmental Biology, University College London; 3 Department of Biological Sciences, HHMI, Columbia University

**Figure 1:**
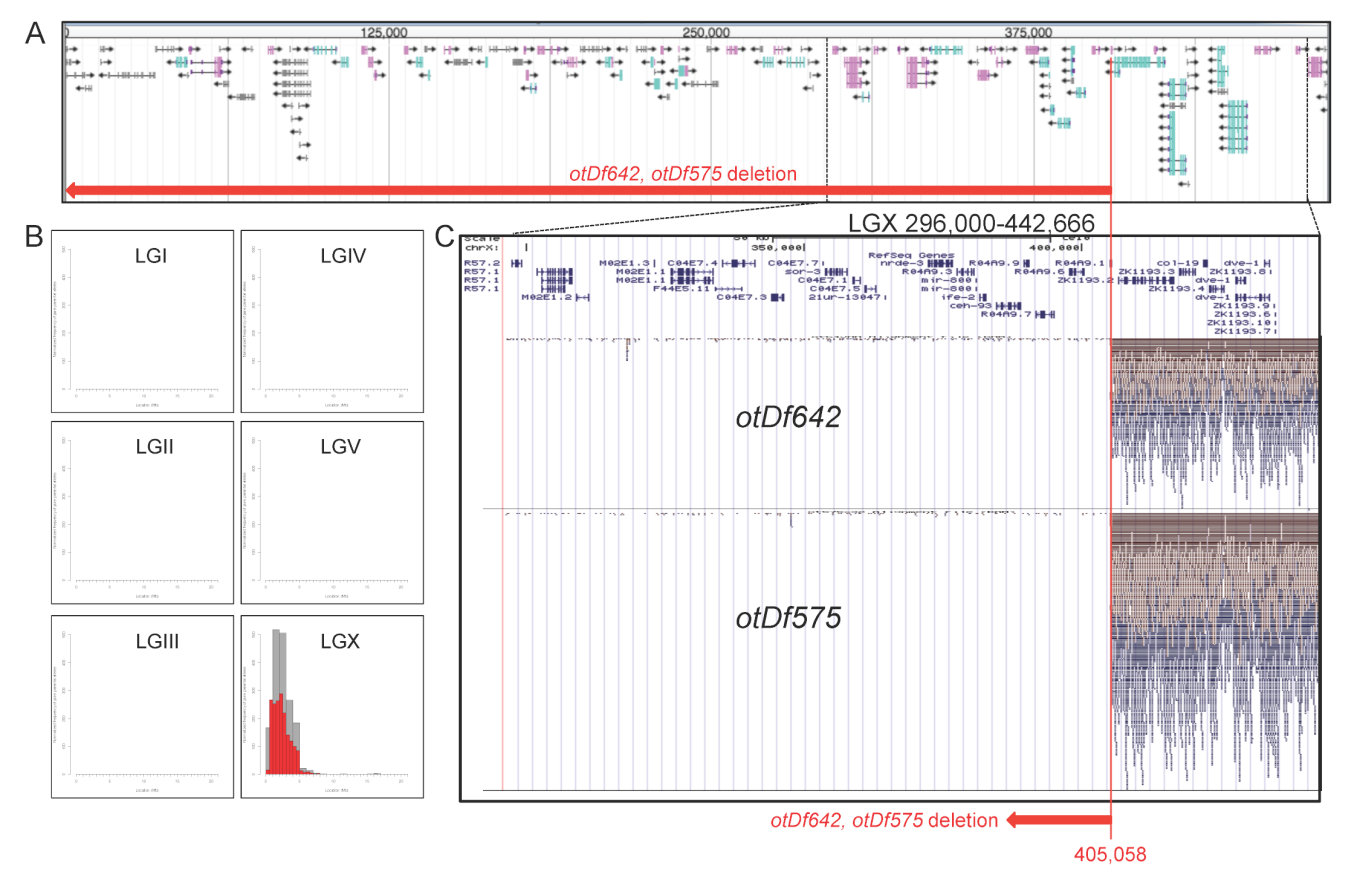
**Mapping and location of *otDf575* and *otDf642.*** (A,C) Sequence of left arm of LGX, with region from 296,000 to 442,666 enlarged (panel C). The deletion breakpoint is at 405,058 and indicated with a red line. Panel C shows aligned Illumina sequencing reads (146,666 bp, from LGX: 296,000-442,666) for *ot642* and *ot575*, as displayed in the UCSC genome browser (Kent *et al*., 2002). A complete absence of coverage, indicative of a deletion, can be observed for both *ot575* and *ot642*. Other strains isolated from the same mutant screen do not show this deletion. (B) *ot642* mapping plot. While *ot575* was mapped by variant discovery mapping (VDM) through crossing with N2 wildtype animals (Method described in Minevich *et al.*, 2014), *ot642* was mapped by WGS-SNP through crossing with Hawaiian polymorphic mapping strain and results are shown in a mapping plot, as previously described (Minevich *et al.*, 2014).

## Description

We are interested in isolating mutants that affect lineage specification in the nervous system of the nematode *C. elegans.* The harsh touch sensory neuron PVD, generated by the postdeirid lineage, can be labeled with two transgenically expressed fluorophores, *ser-2::gfp* (*otIs138* transgene)(Tsalik *et al.*, 2003) and *dop-3:rfp* (*vsIs33* transgene)(Chase *et al*., 2004). Using a *otIs138; vsIs33* double transgenic strain, we screened for EMS-induced mutations in which both markers fail to be expressed in PVD and isolated two strains in which the majority of animals fail to display reporter expression in PVD (*ot642* mutant strain: 56% animals showed no marker expression in PVD expression; *ot575* mutant strain*:* 71% of animals; n=54). *ot575* and *ot642* fail to complement each other. Animals that carry these alleles are viable, fertile and display no obvious morphological abnormalities. Both strains were subjected to Illumina whole genome re-sequencing, one in combination with Hawaiian SNP mapping (*ot642)*(Doitsidou *et al.*, 2010) the other in combination with variant discovery mapping (*ot575)*(Minevich *et al.*, 2012). These two orthogonal mapping approaches revealed that both mutant strains carry the exact same alteration: a loss of 405,058 bp from the extreme left end of the X chromosome (Figure 1). *ot575* and *ot642* were isolated from separate rounds of screens so these mutants were not progeny of the same initial parent. Each of these strains were isolated with the *otIs138[ser2prom3::gfp]* transgene that is also located on chromosome X so it is possible that this transgene somehow contributes to chromosome instability in some way or form.

We confirmed the deletion with PCR primers located within this deletion, which yielded a PCR product from wild-type animals, but not mutant animals. Since the size of the deletions classifies these alleles as deficiencies, we renamed these alleles *otDf575* and *otDf642.*

The deletion in *otDf575* and *otDf642* eliminates 50 protein-coding genes (one of them, *R04A9.1*, which carries the deletion breakpoint, is cut in half), at least 20 pseudogenes and a number of regulatory RNAs, ranging from several 21U RNAs to three miRNA encoding genes, *mir-258.1*, *mir-258.2* and *mir-800*. The protein coding genes lost in these strains include a number of different functional categories, including a number of gene regulatory factors: two transcription factors (*elk-2* and *ceh-93*, a homeobox gene), two SET-domain containing chromatin factors (*set-28*, *set-33*), an argonaute protein (*nrde-3*) and the polycomb-group gene, *sor-3*.

We find that the *sor-3* locus alone is responsible for the PVD mutant phenotype of *otDf575* animals, because *otDf575* fails to complement *sor-3(bp185)* (5/21 *ot575/bp185* transheterozygous animals show PVD loss) and *sor-3(bp185)* homozygotes show the same PVD mutant phenotype as *otDf575* and *otDf642* mutant animals (68% penetrant; n=22). *sor-3* codes for a protein containing an MBT repeat domain that displays methylated histone binding activity and exists in the PcG proteins SCM and Sfmbt in other organisms (Yang et al., 2007). Previous work showed that loss of *sor-3* leads to expression of ectopic dopaminergic and serotonergic male ray neuron fates (Yang *et al.*, 2007). Furthermore, in *sor-3* mutants, the Hox genes *egl-5* and *lin-39* are ectopically expressed outside their usual domain (Yang *et al.*, 2007). Ectopic expression of the *mab-5* gene has previously been shown to eliminate the production of the entire postdeirid lineage (that produces the PVD neuron)(Salser and Kenyon, 1996). We hypothesize that *sor-3* mutants affect PVD neuron differentiation via ectopic expression of endogenous *mab-3*.

## Reagents

**Reagents**

*OH9716 *otDf575; otIs138; vsIs33*

OH9900 otIs138; vsIs33

*OH10091 *otDf642; otIs138; vsIs33*

*Strains will be available at the CGC.
