# Corrigendum: Nibbling 405 kb off the X: Viable deletion alleles eliminating 50 protein coding genes, including a chromatin factor involved in neuronal development

**DOI:** 10.17912/micropub.biology.000206

**Published:** 2019-12-23

## Description

For Minevich, G; Bernstein, A; Mei, K; Poole, RJ; Hobert, O (2019). Nibbling 405 kb off the X: Viable deletion alleles eliminating 50 protein coding genes, including a chromatin factor involved in neuronal development. microPublication Biology. 10.17912/micropub.biology.000187

vsIs33 is incorrectly reported in the following sentence: “The harsh touch sensory neuron PVD, generated by the postdeirid lineage, can been labeled with two transgenically expressed fluorophores, *ser-2*::gfp (*otIs138* transgene)(Tsalik et al., 2003) and *dat-1*:rfp (*vsIs33* transgene) (Chase et al., 2004).”

Authors have corrected the genotype of *vsIs33* to [*dop-3*::rfp]. In addition sentence has been corrected.

The sentence now reads:

“The harsh touch sensory neuron PVD, generated by the postdeirid lineage, can be labeled with two transgenically expressed fluorophores, *ser-2*::gfp (*otIs138* transgene)(Tsalik et al., 2003) and *dop-3*:rfp (*vsIs33* transgene) (Chase et al., 2004).

These errors have been corrected online as well as in future PDF versions of this article.

